# High-status individuals are held to higher ethical standards

**DOI:** 10.1038/s41598-023-42204-z

**Published:** 2023-09-13

**Authors:** Stefan T. Trautmann, Xianghong Wang, Yijie Wang, Yilong Xu

**Affiliations:** 1 Alfred-Weber-Institute for Economics, University of Heidelberg, Bergheimer Str. 58, 69115 Heidelberg, Germany; 2 School of Economics, Renmin University of China, Zhongguancun Str. 59, Beijing, 100872 China; 3 Institute of Governance, Shandong University, Binhai Str. 72, Tsingtao, 266237 China; 4 Utrecht School of Economics, Utrecht University, Kriekenpitplein 21-22, 3584 EC Utrecht, The Netherlands

**Keywords:** Human behaviour, Social evolution

## Abstract

Although there is evidence for the generosity of high-status individuals, there seems to be a strong perception that the elites are selfish and contribute little to others’ welfare, and even less so than poorer people. We argue that this perception may derive from a gap between normative and empirical expectations regarding the behavior of the elites. Using large-scale survey experiments, we show that high-status individuals are held to higher ethical standards in both the US and China, and that there is a strong income gradient in normatively expected generosity. We also present evidence for a gap between people’s normative expectations of how the rich should behave, and their empirical expectations of how they actually do: empirical expectations are generally lower than both normative expectations and actual giving.

## Introduction

A puzzling pattern emerges from the literature on the relationship between ethical behavior and socioeconomic status (SES), and its reception in the public discourse. On the one hand, there seems to be the perception that the rich and successful have the tendency to behave unethically and selfish^1, 2^. Reports on the missteps of some member of the elite often attract much media attention. The same is true for scientific results claiming to show that high-SES individual have low ethical standards. On the other hand, there is converging evidence that high-status individuals often behave equally or more ethical, social, and generous than low-status individuals^3–12^. Such findings are often received with surprise or misbelief. Alternatively, the reaction to the observation of generous behavior by the rich is to suggest that they *should* be more generous, as they do have more resources^13^. Starting from this often-heard normative claim, we argue that the apparent gap between the public perception and interpretation of the ethics of members of the elite, and their actual behavior, may be caused by higher, if not inflated demands on the ethics of these high-SES individuals. In particular, we hypothesize that normative expectations regarding the behavior of the rich (what they should do) exceed empirical expectations regarding their behavior (what people believe they actually do). The gap between normative and empirical expectations about the rich may lead to the perception that they do not do enough, given their advantageous personal situation, which may then lead to the perception that they contribute little in absolute terms. Importantly, if such a gap exists, pessimistic views of the elite may emerge even if empirical expectations regarding their behavior are well calibrated, and not particularly pessimistic in itself. Such a pattern of expectations, if widely held in the population, may harm social coherence by eroding trust in important political and social institutions, which are typically designed and dominated by the elites^14^. Moreover, a perceived violation of normative expectations regarding the behavior of the rich may also lead to negative contagious behavioral effects, justifying unethical and selfish behavior more widely in the population^13^. It would necessitate a public discourse about the different social classes’ appropriate contribution to the common good.

In this paper we proceed to test the hypothesized pattern of expectations as follows. In Study 1, we present survey data from the US and China, testing how people assess a variety of unethical behaviors committed by either a high-SES or a low-SES individual. That is, we test if people hold higher normative expectations regarding high-SES individuals. US data are collected through Amazon Mechanical Turk, Chinese data are collected through the Shandong General Social Survey. The behaviors we study regard the treatment of a false bank transfer and welfare/tax fraud (petty vs. severe) as examples of financial misbehavior, and the abuse of an emergency lane and trafficking of endangered species as non-financial behaviors. We elicit three assessments regarding these behaviors if committed by either high- or low-SES individuals, operationalizing SES in terms of income. First, we elicit how acceptable the behavior is in general. Second, we elicit how harmful the behavior is perceived for society. And third, we elicit how diagnostic people perceive unethical behavior in one domain for unethical behavior in other domains of life. Assessments for high-SES and low-SES individuals’ behavior is studied in a between-subjects design. Using demographic information on the survey respondents, we also distinguish between judgments by high- versus low-SES respondents.

Study 2 focusses on normative expectations regarding generosity. In a simple dictator allocation game, we elicit what survey participants consider an appropriate transfer from the allocator to the recipient. We elicit these normative expectations for allocator income levels ranging from $10,000 to $1 M, for two different income levels of the recipient ($40,000 or $80,000), in a full between-subjects design. The design allows us to test if the transfer by the allocator that is considered appropriate increases by allocator income, and depends on the income position relative to the recipient.

While Study 1 and 2 test whether there are differences in normative expectations regarding the rich and the poor, Study 3 aims to directly compare empirical and normative expectations regarding a specific behavior of the rich. To this end, we build on an experimental measurement of the generosity of millionaires in a simple allocation decision in a previous study^7^, and elicit respondents’ normative and empirical expectations regarding the allocations chosen by the millionaires in that experiment. We compare respondents’ empirical expectations to the true behavior of the millionaires to assess whether they hold pessimistic views about the millionaires’ actual behavior. We compare respondents’ empirical expectations to their normative expectations in a between-subject design, to test whether the hypothesized gap between the desired and the perceived behavior of the rich is prevalent for the generosity task studied.

## Results

### Study 1: ethical demands on the rich and the poor

A total of N = 2110 respondents participated in the US survey, conducted through Amazon Mechanical Turk (MTurk). Each respondent participated in exactly one of ten conditions that result from crossing five unethical behaviors with two status levels. For each condition, respondents read the vignette describing the unethical behavior committed by either the high-SES or the low-SES individual, and then assessed the behavior with regard to its acceptability, perceived harmfulness for society, and diagnosticity for unethical behavior in other contexts. We assess these views with simple single-item survey questions which we considered most suitable for the cross-cultural nature of the study. Table 1 shows results. Pooling all behaviors, we find that respondents consider unethical behavior less acceptable, more harmful, and more diagnostic for more widely unethical behavior, if committed by high-status individuals. With overall mean acceptability, harmfulness, and diagnosticity scores of 2.66, 6.65, and 2.90 for high-status, and 3.25, 5.83, and 3.72 for low-status individuals, we observe overall low acceptability of unethical behavior, where people find behaviors modestly harmful for society and rather diagnostic for behavior in general. We next subdivide the data by unethical behavior and by the social status of the *respondents* assessing the vignette. We refer to the respondents who self-reported having below (above) and far below (above) average family income in the area they live as the low-status (resp. high-status) respondents. Results show that effects are predominantly driven by (1) low-status respondents and (2) unethical behavior in the financial domain, that is, by assessments of welfare/tax fraud and the non-reporting of a misdirected bank transfer. Note that the vignettes in the financial domain specify very different settings in terms of the monetary impact of the unethical actions of the low-SES and the high-SES individual. That makes it potentially difficult to directly compare these scenarios. What is important for our research focus is that across the different scenarios, we find consistent associations of status with the measured views.

**Table 1:** Assessments of unethical behaviors, US, MTurk.

Respondent	SES-condition	Bank	Welfare/tax fraud (low)	Welfare/tax fraud (high)	Emergency lane	Endangered animals	Overall
Acceptability of behavior (0 =least acceptable to 10 most acceptable)							
All data	High status	2.97 (0.22)	2.74 (0.22)	2.25 (0.22)	2.69 (0.20)	2.63 (0.24)	2.66 (0.10)
	Low status	3.28 (0.23)	4.17*** (0.24)	3.29*** (0.23)	2.73 (0.19)	2.77 (0.22)	3.25*** (0.10)
High family income	High status	3.20 (0.51)	3.31 (0.54)	2.33 (0.57)	3.41 (0.53)	3.10 (0.55)	3.15 (0.24)
	Low status	2.72 (0.58)	4.20 (0.52)	3.43 (0.50)	2.55 (0.44)	2.67 (0.51)	3.09 (0.24)
Low family income	High status	2.26 (0.35)	3.00 (0.44)	1.92 (0.34)	2.77 (0.35)	3.00 (0.47)	2.58 (0.17)
	Low status	3.68** (0.46)	4.94*** (0.49)	3.93*** (0.45)	2.64 (0.39)	2.70 (0.37)	3.54*** (0.20)
Harmfulness of behavior (0 = least harmful to 10 = most harmful)							
All data	High status	5.91 (0.20)	6.25(0.23)	6.89 (0.20)	6.86 (0.20)	7.44 (0.21)	6.65 (0.09)
	Low status	5.03 (0.22)	5.01***(0.23)	5.58*** (0.23)	6.35* (0.20)	7.16 (0.20)	5.83*** (0.10)
High family income	High status	5.61 (0.47)	6.66 (0.53)	7.61 (0.38)	7.30 (0.48)	7.46 (0.42)	6.93 (0.20)
	Low status	6.00 (0.58)	5.45 (0.50)	5.89*** (0.48)	6.92 (0.49)	7.62 (0.46)	6.34* (0.23)
Low family income	High status	6.00 (0.35)	6.16 (0.46)	6.70 (0.38)	6.18 (0.36)	6.85 (0.42)	6.37 (0.18)
	Low status	4.71** (0.42)	4.39*** (0.46)	5.16*** (0.44)	6.09 (0.39)	6.95 (0.34)	5.52*** (0.19)
Diagnosticity of unethical behavior for other situations (0 = very unethical to 10 = very ethical, in other situations)							
All data	High status	3.09 (0.20)	2.97 (0.21)	2.58 (0.21)	2.80 (0.19)	3.08 (0.21)	2.90 (0.09)
	Low status	3.74** (0.20)	4.16*** (0.20)	3.74*** (0.21)	3.61*** (0.21)	3.38 (0.20)	3.72*** (0.09)
High family income	High status	3.36 (0.46)	4.13 (0.59)	2.44 (0.51)	3.60 (0.52)	3.62 (0.53)	3.43 (0.24)
	Low status	3.08 (0.52)	4.18 (0.47)	3.76* (0.45)	3.30 (0.47)	3.64 (0.52)	3.62 (0.22)
Low family income	High status	2.58 (0.30)	2.87 (0.36)	2.21 (0.35)	2.59 (0.32)	3.35 (0.42)	2.70 (0.16)
	Low status	4.22*** (0.38)	4.17** (0.42)	4.04*** (0.39)	3.13 (0.40)	3.68 (0.34)	3.85*** (0.17)

Column *Respondent* indicates status of survey respondent, based on self-reported family financial situation on a 5-point scale. Those below (above) the midpoint of 3 are categorized into the Low (High) Family Income group. Column *SES-Condition* indicates social status of person committing unethical behavior in the vignette. *, **, *** at the entries for Low Status indicates that these values differ from those for High Status in the same cell, at the 10%, 5%, and 1% significance level, two-sided t-tests. Standard errors are reported in parenthesis.

Interestingly, low-status respondents do not hold overall higher or lower assessments than high-status respondents do (who distinguish little by whom an unethical behavior is committed). They rather perceive unethical acts by high-SES individual as more problematic, and those by low-SES individuals as less problematic, than high status respondents. For example, high-status respondents give mean acceptability scores of 3.15 and 3.09 for high- and low-SES individuals’ behavior, while low-status respondents give mean acceptability scores of 2.58 and 3.54 for high- and low-SES individuals’ behavior.

A total of N = 923 respondents participated in the Chinese survey, conducted through the Shandong General Social Survey administered by Shandong University. The setup of the study and wording of all questions was exactly identical to the US sample conducted through MTurk. However, in contrast to the MTurk sample, the Shandong survey aims to be representative of the population of Shandong Province in China (~ 100 m inhabitants). Table 2 shows the results. For the assessments of the acceptability and the harmfulness of the unethical behaviors we find the same pattern as in the US: acts are perceived less acceptable and more harmful if committed by high-SES individuals. Again, the effects are driven by lower-status respondents, and especially the financial behaviors for the case of perceived harmfulness. In contrast to the US data, Chinese respondents do not distinguish between high- and low-SES individuals in their assessment of the diagnosticity of the unethical behaviors for how a person will behave in other situations. In general, the Chinese respondents considered the activities even less acceptable (1.88 vs. 2.96, 95% CI for the difference: [− 1.31, − 0.83], N = 3,026, *p* < 0.0001, two-sided t-test), more harmful (7.30 vs. 6.24, 95% CI for the difference: [0.82, 1.30], N = 3024, *p* < 0.0001, two-sided t-test), and more diagnostic for general unethical behavior (2.57 vs. 3.32, 95% CI for the difference: [− 0.97, − 0.52], N = 3017, *p* < 0.0001, two-sided t-test) than the US sample. Despite these relevant differences, and across two very different cultural backgrounds, Study 1 thus shows a universal pattern to hold high-SES individuals to higher ethical standards than low-SES individuals.

**Table 2:** Assessments of unethical behaviors, China, Shandong General Social Survey.

Respondent	SES-condition	Bank	Welfare/tax fraud (low)	Welfare/tax fraud (high)	Emergency lane	Endangered animals	Overall
Acceptability of behavior (0 = least acceptable to 10 most acceptable)							
All data	High Status	1.37 (0.25)	1.61 (0.27)	1.66 (0.28)	2.07 (0.29)	1.64 (0.26)	1.68 (0.12)
	Low Status	1.71 (0.28)	2.36* (0.29)	2.05 (0.28)	2.56 (0.29)	1.77 (0.27)	2.09** (0.13)
High family income	High Status	1.40 (1.40)	2.17 (1.05)	1.17 (0.75)	1.82 (0.87)	1.86 (0.80)	1.78 (0.44)
	Low Status	0.75 (0.46)	2.70 (1.08)	2.88 (1.30)	3.14 (1.10)	1.73 (0.63)	2.06 (0.39)
Low family income	High Status	1.59 (0.44)	1.13 (0.37)	1.88 (0.46)	1.96 (0.62)	1.66 (0.28)	1.51 (0.20)
	Low Status	2.18 (0.54)	2.00 (0.53)	2.06 (0.41)	2.11 (0.45)	1.82 (0.31)	2.14** (0.22)
Harmfulness of behavior (0 = least harmful to 10 = most harmful)							
All data	High status	7.39 (0.29)	7.64 (0.30)	7.48 (0.27)	7.45 (0.31)	7.40 (0.28)	7.47 (0.13)
	Low status	6.97 (0.34)	6.58** (0.32)	7.04 (0.29)	7.48 (0.27)	7.53 (0.28)	7.12* (0.13)
High family income	High status	8.20 (0.92)	7.25 (1.04)	7.33 (1.02)	7.18 (0.93)	6.14 (1.12)	7.17 (0.46)
	Low status	7.58 (0.90)	6.90 (0.94)	7.13 (1.23)	6.29 (1.06)	7.07 (0.76)	7.06 (0.41)
Low family income	High status	7.71 (0.43)	7.97 (0.51)	7.47 (0.42)	7.48 (0.68)	7.75 (0.42)	7.68 (0.24)
	Low status	5.94** (0.65)	6.38** (0.58)	6.78 (0.46)	7.84 (0.41)	6.92 (0.52)	6.82*** (0.21)
Diagnosticity of unethical behavior for other situations (0 = very unethical to 10 = very ethical, in other situations)							
All data	High status	2.37 (0.28)	2.31 (0.26)	2.40 (0.26)	2.83 (0.26)	2.76 (0.28)	2.54 (0.12)
	Low status	2.09 (0.23)	2.48 (0.24)	2.72 (0.26)	3.00 (0.27)	2.74 (0.24)	2.60 (0.12)
High family income	High status	2.60 (1.60)	2.33 (0.70)	2.17 (1.01)	2.27 (0.70)	5.14 (0.88)	2.80 (0.42)
	Low status	2.08 (0.63)	3.40 (0.82)	2.38 (0.82)	4.29** (0.42)	2.79* (0.83)	2.88 (0.35)
Low family income	High status	2.55 (0.49)	1.81 (0.42)	2.94 (0.42)	2.43 (0.61)	2.93 (0.41)	2.53 (0.21)
	Low status	1.94 (0.39)	2.58 (0.47)	3.25 (0.44)	2.53 (0.40)	3.23 (0.49)	2.68 (0.20)

Column *Respondent* indicates status of survey respondent, based on self-reported family financial situation on a 5-point scale. Those below (above) the midpoint of 3 are categorized into the Low (High) Family Income group. Column *SES-Condition* indicates social status of person committing unethical behavior in the vignette. *, **, *** at the entries for Low Status indicates that these values differ from those for High Status in the same cell, at the 10%, 5%, and 1% significance level, two-sided t-tests. Standard errors are reported in parenthesis.

### Study 2: generosity demand on the rich and the poor

A total of N = 2243 respondents participated in this study, conducted through Amazon Mechanical Turk (MTurk). In this study, we describe a simple dictator game to the respondents and inform them about both the allocator’s and recipient’s annual income. This information is the treatment variation we employ. We then elicit the normative expectation of our respondents regarding the appropriate amount of transfer in the dictator game from the allocator to the recipient, and test whether it is affected by the income of the players. There are 28 conditions in total, each with a different combination of allocator’s and recipient’s income. Specifically, there are 14 levels for allocator’s income and 2 levels for the recipient’s income. We implemented a between-subject design such that each respondent participated in exactly one condition only. The current design allows us to analyze individuals’ normative expectation when the allocator’s income is higher, lower, or equal to the recipient’s income, for two distinct levels of the recipient’s income.

**Figure 1:**
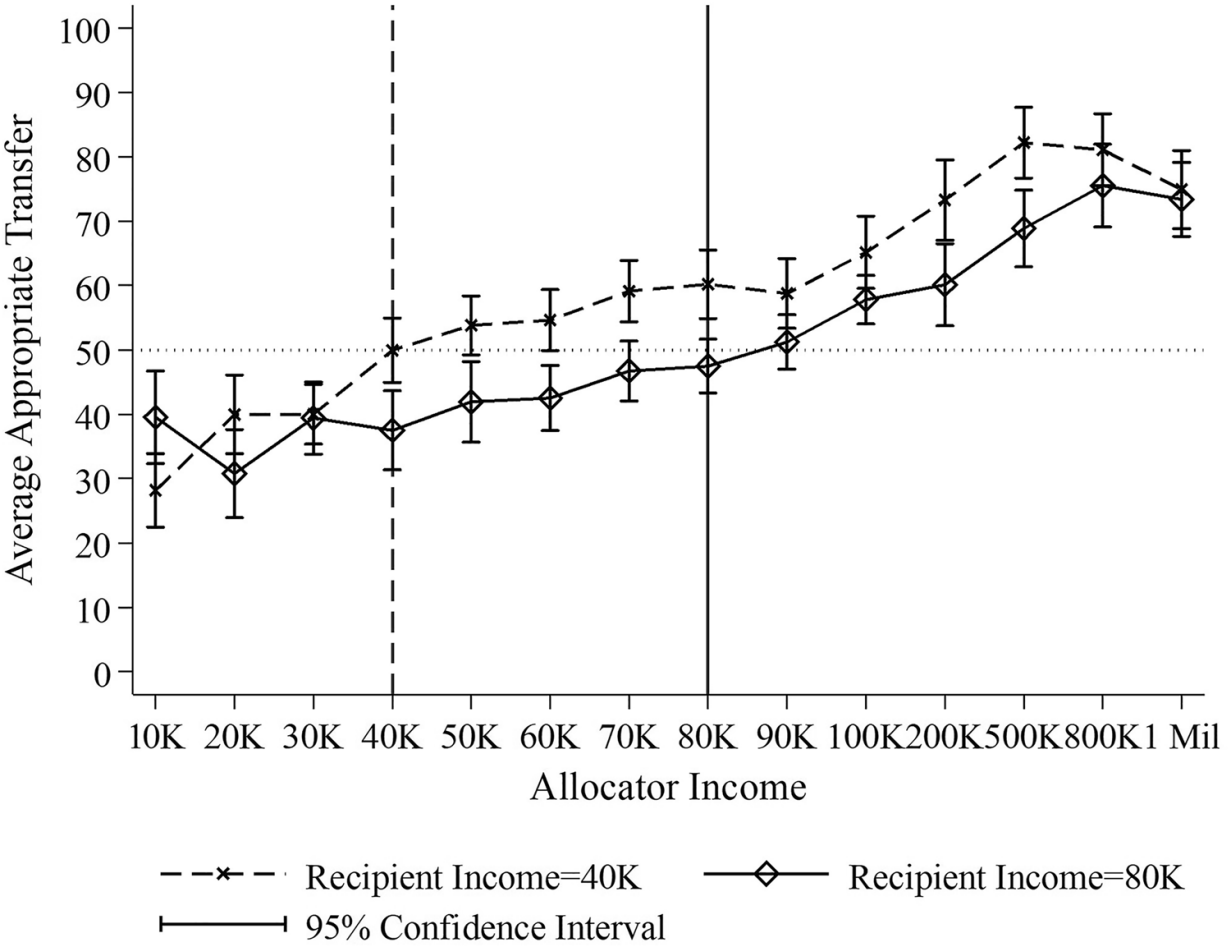
Average Appropriate Transfer by income levels. The figure shows the transfer considered appropriate by the respondents, depending on the income of the allocator and the recipient.

Figure 1 depicts the results. The vertical axis shows the average transfer that respondents find appropriate and the horizontal axis indicates the allocator’s income. We see a clear trend that the higher the allocator’s income is, the more people demand her to transfer. This holds true both when the recipient’s annual income is $40,000 (the dashed line) and $80,000 (the solid line). When the allocator has the same income as the recipient, the average appropriate transfer is approximately $50, regardless of the income of the recipient (the appropriate transfer value is not significantly different from $50 at the 5% significance level, two-sided exact Wilcoxon signed-rank tests for both recipient’s income levels. When both earn $80,000 annually, the 95% CI of the appropriate transfer is $[43.25, 51.72], N = 80; when both earn $40,000, the 95% CI is $[44.87, 55.03], N = 80). When the allocator’s income is lower than the recipient’s income, respondents think that the allocator who has lower income can give less than $50. On the other hand, when the allocator’s income increases, respondents find a higher transfer more appropriate. This observation holds true for both income levels of the recipients.

The average transfers deemed appropriate are summarized in Table 3. The left and right panels show respondents’ normative expectations when the recipient’s income is $40,000 and $80,000 respectively. Considering all respondents, we see that allocators who earn less (more) than the recipients are expected to give statistically significantly less (more) than the allocators who earn the same income as the recipient, for both recipient income levels. Moreover, we also observe that when the allocators’ income is between $40,000 to $80,000 per year, they are required to transfer on average $55.85 (N = 240) when the recipient’s income is $40,000 and $43.74 (N = 238) when the recipient’s income is $80,000 (95% CI for the difference: [7.99, 16.25], N = 478, *p* < 0.0001 two-sided t-test). Thus, the higher the allocator’s absolute and relative (to the recipient) income is, the higher the demand on her generosity.

**Table 3:** Average appropriate transfer by income levels.

Respondent	Recipient’s income: $40 K			Recipient’s income: $80 K		
	Allocator’s Income < $40 K	Allocator’s Income = $40 K	Allocator’s Income > $40 K	Allocator’s Income < $80 K	Allocator’s Income = $80 K	Allocator’s Income > $80 K
All	$35.98^a^ ($1.66)	$49.95^b^ ($2.55)	$66.30^c^ ($0.94)	$39.76^a^ ($1.17)	$47.49^b^ ($2.13)	$64.46^c^ ($1.21)
High family income	$38.96^a^ ($4.09)	$51.62^a^ ($6.26)	$64.99^b^ ($2.04)	$48.61^a,###^ ($2.63)	$43.89^a^ ($6.06)	$62.44^b,#^ ($2.67)
Low family income	$32.50^a^ ($2.73)	$51.91^b^ ($4.04)	$69.70^c^ ($1.87)	$33.09^a^ ($1.81)	$50.25^b^ ($2.52)	$69.22^c^ ($2.19)

Column *Respondent* indicates status of survey respondent, based on self-reported family financial situation on a 5-point scale. Those below (above) the midpoint of 3 are categorized into the Low (High) Family Income group. Within each row and panel, entries with identical superscripts are not statistically significantly different from each other at the 5% significance level, two-sided Mann Whitney U tests. Results are robust if two-sided t-tests are used instead. Entries that are significantly different from each other do not share the same letter. Standard errors are reported in parenthesis. ###, ##, # indicate the level of significance of 0.01, 0.05, 0.1 when testing the differences between the answers given by the high family income respondent and the low family income respondents.

Next, we show answers separately by the family income of the respondent. We refer to the respondents who self-reported having below (above) and far below (above) average family income in the area they live as the low-status (resp. high-status) respondents. We see that the general pattern described above obtains for both groups, but is more pronounced for the low-status respondents: compared to high-status respondents, low status respondents believe that lower-income allocators need to give less, and higher-income allocators need to give more. The pattern can be observed in both panels, but is statistically significant only for the high-income recipient (right panel). This pattern is also confirmed in multi variate regressions of the appropriate transfer amount run on the allocator income and its interaction with respondent status, see Supplementary Table 1. The result replicates the finding in Study 1 that the higher demand on the ethics of the rich is strongly driven by lower income respondents.

It may be argued that the effect of relative income on the transfer considered appropriate is a simple expression of inequality aversion. However, note that even for the smallest differences considered (i.e., $10,000), a strict focus on inequality would suggest giving the full amount of $100 to the recipient if the allocator has higher income, and keeping the full amount if the allocator has lower income. Clearly, this is not the case: multiple equity norms compete in the current setting. To better understand the respondents’ motivations, we analyze the justifications provided by the respondents when submitted their answers regarding the appropriate transfer. We construct a variable *Motivation*, coded as 1 if the respondent expresses only inequality concerns; 0 if she expresses both fairness and inequality concerns; and − 1 if she expresses only fairness concerns. Supplementary Fig. 1 (based on Fig. 1) shows that the categorization is meaningful: respondents scored as inequality-concerned reveal a strong allocator-income gradient for the appropriate transfer, while the pattern is basically flat and close to the equal split of the money for those scored as fairness concerned. Supplementary Fig. 2 shows the relationship between respondents’ motivations and allocator income. It plots the fitted regression values from a regressing of *Motivation* on allocator’s income and income squared. The figure shows that for both recipient income levels, the greater the income differences are, the more respondents are concerned about inequality. When incomes are similar, fairness concerns dominate. That is, income levels affect which norm is perceived as more compelling, and the norm is then associated with the transfer considered appropriate by the respondent. Different norms apply to different individuals, depending on their status.

### Study 3: normative versus empirical expectations about the generosity of the rich

A total of N = 837 respondents participated in either of two between-subjects conditions of Study 3, conducted through Amazon Mechanical Turk. In the first condition we elicit the respondents’ empirical expectations regarding the actual giving behavior of millionaires in a simple allocation task that was observed in a previous study by Smeets and coauthors^7^. In the second condition we elicit the respondents’ normative expectations regarding the giving by millionaires in that same task. Each condition had two between-subject sub-conditions, distinguished by whether the millionaires allocated money between themselves and another millionaire (“rich partner”), or between themselves and another low-income person (“poor partner”). In all conditions, respondents read a description of the experimental task that the millionaires were presented with in the previous study. The key parameters of the situation are as follows^7^: Millionaires were customers of a bank cooperating in the study, each having liquid wealth larger than €1 m. Low-income recipients were people with a gross income of less than €12.500. The millionaires had to distribute €100 between themselves and another person (either rich or poor). Moreover, in all conditions, respondents were informed that in this type of allocation tasks, across a large number of studies with participants who are neither particularly rich or poor, the average giving to the other person is 28%^15^.

Respondents then answered two questions, either regarding the actual, or regarding the desirable giving by the millionaires in their condition (empirical/normative X rich partner/poor partner). First, they indicated whether they think that millionaires gave (should give) more or less than the “typical” giving of 28% of the available amount. Second, conditional on their first decision, they indicated the precise number how much they thought the millionaires gave on average (should give), out of the €100.

**Figure 2:**
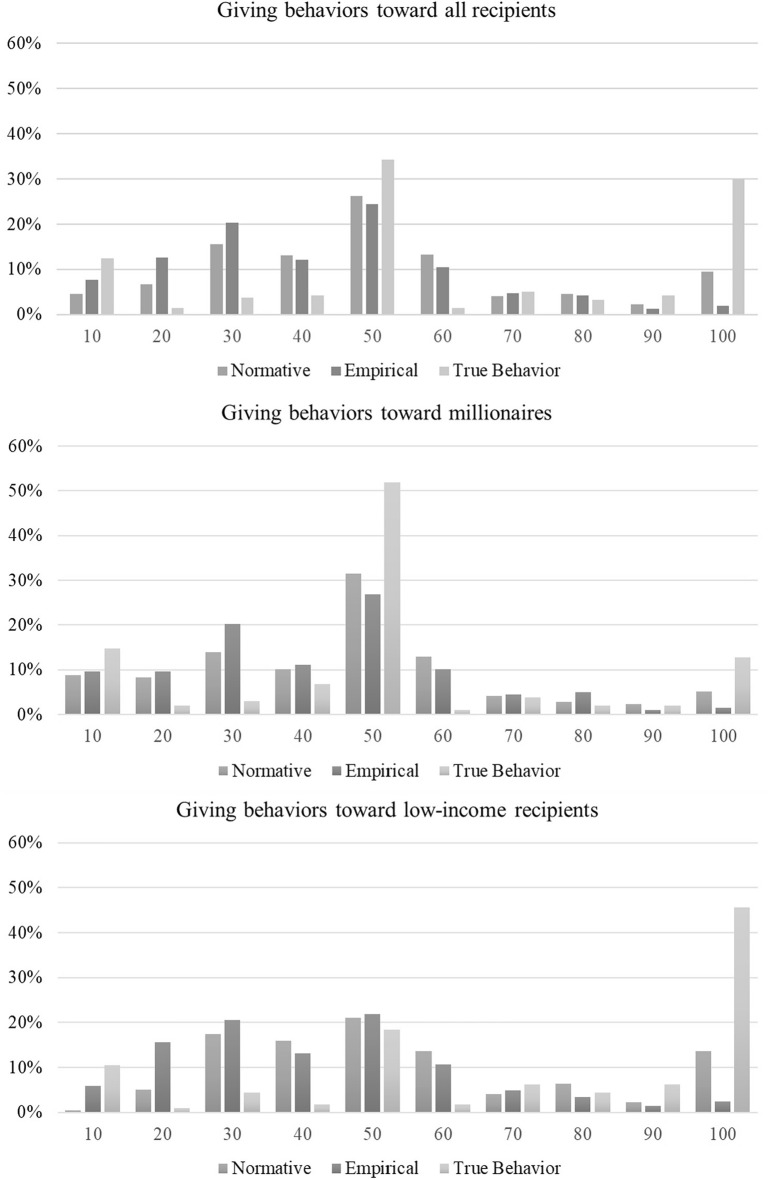
Normative and empirical expectations regarding millionaires’ giving. The figure shows the distribution of giving by millionaires (“true behavior”) to all other recipients, to other millionaires, and to low income recipients. It also shows the distribution of normative and empirical expectations for each of these cases.

Table 4 shows the results. We find that normative expectations are significantly higher than empirical expectations for both, the share of millionaires who give more than typical allocators, and for the exact offers in euros. These effects are mainly driven by the conditions where the millionaire allocates money to a low-income partner. Here the respondents have substantially larger demands on the millionaires’ generosity than what they think millionaires actually give. The gap is smaller and insignificant (but still positive in direction) for the rich partners. This is caused by lower normative expectations, while empirical expectations are identical to the case of low-income partners. Interestingly, both empirical expectations and normative expectations, are substantially lower than the true giving by the millionaires. Figure 2 depicts the full distribution of expected and true offers. A pattern that sticks out is the severe underestimation of millionaires giving the full amount to a poor partner: about 15% of the respondents consider that this is what the millionaires should do, while few think they actually gave that much. However, in the experiment, almost half of the millionaires indeed gave the full amount to the poor partner.

**Table 4:** Normative and empirical expectations regarding millionaires’ giving.

	Share of respondents: offer > 28%			Offer in euro		
	Normative	Empirical	True Decisions	Normative	Empirical	True Decisions
Rich partner	77.31%^a,b^ (0.03)	71.57%^a^ (0.03)	83.33%^b^ (0.04)	44.45^a^ (1.58)	40.18^a^ (1.46)	49.61^b^ (2.76)
Poor partner	88.58%^a^ (0.02)	70.24%^b^ (0.03)	88.60%^a^ (0.03)	53.02^a^ (1.66)	39.98^b^ (1.47)	71.40^c^ (3.17)
All	82.99%^a^ (0.02)	70.90%^b^ (0.02)	86.11%^a^ (0.02)	48.77^a^ (1.16)	40.08^b^ (1.03)	61.11^c^ (2.24)

Within each row and panel, entries with identical superscripts are not statistically significantly different from each other at the 1% significance level, two-sided. Entries that are significantly different from each other do not share the same letter. When comparing the share of respondents who give more than 28%, normative and empirical expectations are compared using test of proportions; and expectations are compared to true shares using binomial tests. When comparing exact offers, two-sided t-tests are employed. Standard errors are reported in parenthesis.

## Discussion

In two surveys with two populations of very different cultural and economic background, Study 1 finds clear evidence that higher social status implies higher demands on a person’s ethical behavior**—***noblesse oblige*. A second study further demonstrates that higher income individuals are expected to be more generous, while the less well-off are allowed to be more selfish, in a simple allocation game. In both Study 1 and 2, the pattern is strongly driven by survey respondents who are of lower socio-economic status. The data suggest that different norms apply to different individuals depending on their status.

In a third study, we use data on real millionaire’s generosity in the same type of allocation game. We find that empirical expectations regarding generosity are lower than what people think these rich individuals should give. This is especially true in interactions of the rich with the poor. We argue that the common perception of the elites as being selfish and greedy may be driven less by an actual lack of generosity and ethical behavior, but rather by the gap between high normative expectations and pessimistic empirical expectations. People perceive that the rich do not do enough for the common good, given their favorable position in society. This perception may then evolve into an interpretation of them doing too little in absolute terms. Because of the potentially harmful effects on the trust in societal institutions if the economic and political elites are perceived as selfish and unethical, and the apparent potential for contagion of perceived unethical behavior by the rich^13^, socio-economic elites should be aware that they are held to higher standards. Their ethical lapses are considered as more harmful than those by less well-off individuals, and are seen as diagnostic for misbehavior in other domains. While the elites often hold meritocratic views, believing to deserve their position^16^, society at large may not agree, and may require above-average ethical standards to come with above-average socio-economic position.

There are potential limitations of our study, implying future research directions. Our U.S. samples are recruited from the MTurk platform. Although the platform is widely used and provides a demographically more diverse group of respondents than student population convenience samples, it is still not a representative sample and generalization may not be warranted. Extending the current framework to representative samples can inform about the distribution of the observed pattern of views across the current population in the U.S., and possibly other countries. Relatedly, an interesting question for future research concerns the question whether the observed pattern of normative and empirical expectations is moderated by people’s perception of how wealthy high-status individuals attained their favorable positions. Despite our converging evidence across two different cultural backgrounds, such perceptions may differ more widely across countries, and potentially affect the observed *noblesse-oblige* effect identified in the current study.

## Methods

### Ethics declaration

All experiments reported in this paper were conducted according to the rules and regulations for good academic practice of the University of Heidelberg (https://www.uni-heidelberg.de/en/university/about-the-university/good-academic-practice), defined by the university’s Commission for the Safeguarding of Good Academic Practice (Kommission zur Sicherung guter wissenschaftlicher Praxis und zum Umgang mit Fehlverhalten in der Wissenschaft, Universitätsverwaltung, Dezernat Recht und Gremien, Seminarstr. 2, 69117 Heidelberg, Germany). The Commission waives the need for ethics approval for non-medical studies like the surveys presented in the current paper (https://www.uni-heidelberg.de/en/documents/rules-for-safeguarding-good-academic-practice-and-handling-academic-misconduct/download). All participants were above 18 years old and provided explicit written consent to participating on the first screen of the survey.

### Study 1

#### Sample and design

We conduct our first study with U.S. participants on the Amazon Mechanical Turk (MTurk) platform and Chinese participants of the Shandong General Social Survey (SGSS). MTurk has been increasingly used as a platform to conduct social science surveys, as it offers convenient access to a highly diverse population in the U.S.^17–20^. The SGSS aims to serve as a representative sample of the Shandong Province in China, covering 203 communities in the province (with a population of about 100 million).

For the U.S. sample, we posted our study on MTurk including a short description of the task, the task requirement, as well as the expected payment for completing the task. After accepting the task, participants were redirected to our survey that is programmed in oTree^21^. We set a restriction that allows only U.S. residents to enter the survey. We also added a CAPTCHA in the welcome page to prevent bots from entering our study. A total of 2,110 respondents finished our survey, of which 440 were randomly assigned to the Bank condition, 413 to the petty welfare/tax fraud condition, 420 to the severe welfare/tax fraud condition, 432 to the emergency lane condition, and 405 to the endangered animal condition. About half of the participants in each condition were confronted with unethical behavior of a high-SES and the other half with that of a low-SES individual. For the welfare/tax conditions, the high-SES behavior regarded tax fraud, the low-SES behavior regarded welfare fraud. For each scenario, we also separately present results for respondents whose household income is higher or lower than the average, according to self-report with the neutral category being average income. For the relatively high (low) household income respondents, there were 80 (127) in the bank condition; 76 (116) in the petty welfare/tax fraud condition; 82 (136) in the severe welfare/tax fraud condition, 77 (127) in the emergency lane condition, and 89 (129) in the endangered animal condition.

For the SGSS, our vignettes were included as one of the sections in the SGSS’s general surveys. The surveys were conducted in person at the respondents’ home by 182 trained investigators in 2019. In total 923 individuals participated in our survey: 185 of them were randomly assigned to the Bank condition, 184 to the petty welfare/tax fraud condition, 176 to the severe welfare/tax fraud condition, 193 to the emergency lane condition, and 185 to the endangered animal condition. Again, we also present results by household income of the respondents for each scenario. For the relatively high (low) household income respondents, there were 17 (68) in the bank condition, 22 (57) in the petty welfare/tax fraud condition; 14 (65) in the severe welfare/tax fraud condition, 18 (62) in the emergency lane condition, and 22 (54) in the endangered animal condition.

#### Vignettes

In what follows, we list the vignettes used in the MTurk study. The SGSS study uses exactly the same wording, translated to Chinese and using Chinese Yuan instead of dollar amounts (using the exchange rate of $1 = 7.2 at the time of survey, and rounded to round Yuan amounts). The translated scripts are available upon request.

##### Condition: bank

Consider the following setting: A low-skilled worker [successful businessman] who does not check his private bank account very regularly discovers that he incorrectly received a payment of $700 some weeks ago. Given that the transaction had not yet been discovered and reversed, he decides to not report the payment, hoping that it may never be discovered.

##### Conditions: petty [severe] welfare/tax fraud

*For low-SES:* Consider the following setting: A low-skilled worker lost his job some time ago. He, therefore, started an eBay store. He has been earning profits that are similar to his previous wage. Yet, he did not report his new income to the government and kept collecting subsistence allowance that accumulated to $1000 [$10,000]. Since the government has not discovered the fact the he is ineligible for the allowance, he decides not to voluntarily report his income earned from the online store.

*For high-SES:* Consider the following setting: A successful businessman has some overseas business that he has not reported to the tax authority. From the activities abroad, he has been earning profits that he should pay tax for, with the tax owed accumulated to $1000 [$700,000]. Given that the government has not discovered his overseas activities, he decides not to voluntarily report his income.

##### Condition: emergency lane

Consider the following setting: A low-skilled worker who has to show up at a construction site [A successful businessman who has a business meeting] outside of town is stuck in a traffic jam caused by an accident. Because he wants to show up on time, he decides to pass the accident site on the emergency lane, which is strictly reserved for ambulance and police. Because emergency services are busy at the accident site, he is not stopped and punished, and arrives at the construction site (the meeting) on time.

##### Condition: endangered animal

Consider the following setting: A low-skilled worker has recently been sent to an African country by his company. [A successful businessman has recently travelled for business to an African country.] He finds out that he can buy rhino horn powder on the black market there, which is considered an important cure-all by his family. Since he knows that it is forbidden to buy rhino horn in his home country, as rhinos are classified as an endangered animal, he uses this chance to buy this powder to take home.

*All conditions share the same questionnaire:* 
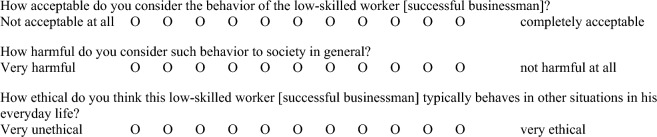


### Study 2

#### Sample and design

The study recruited 2243 U.S. participants through MTurk, following the same recruitment procedure as in Study 1. We described a dictator game to the respondents, in which an allocator has to distribute $100 between herself and another person, the recipient. We informed them about the respective incomes of the allocator and the recipient. Respondents were randomly assigned to one of 28 conditions that differ by allocator and recipient income, with about 80 respondents per condition. We have consulted labor market data from the U.S. Bureau of Labor Statistics and the United States Consensus Bureau for the income distribution in the United States. The allocator’s income in our experiment ranged from $10,000 per year to $1,000,000 per year. As the vast majority (roughly 87%) of the U.S. population earned less than $100,000 per year according to the 2019 U.S. Consensus Bureau^22^, we adopted fine steps of $10,000 for annual income below $100,000. We then included fewer steps towards the annual income of one million dollars, using $200,000, $500,000, $800,000, and $1,000,000. For the recipient’s annual income, we employ two levels: $40,000 and $80,000. According to the Bureau of Labor Statistics^23^, these correspond to the salaries for a typical individual with a high school diploma and a Master’s degree respectively.

For each condition, we asked our respondents to indicate what amount of the $100 endowment the allocator should transfer to the recipient, and to provide some motivations for their answer (it was possible to leave the entry for the motivation empty). The background information regarding the income of the allocator and the recipient is available to the respondents throughout the decision process. Comprehension questions were included to make sure respondents only proceed to the decision stage if they understood the key parameters of the game.

#### Instructions

The instructions of the survey experiment read as follows.

Please consider the following situation carefully and let us know what ***you*** think is the most appropriate behavior of the person in this context.

Suppose that a group of researchers studying individuals’ social behavior has recruited a person with an annual gross income of $Allocator’s Income [$10,000–100,000 (with a 10 K step), $200,000, $500,000, $800,000, $1,000,000] to participate in a study. Let us call this person the allocator.

The allocator is randomly matched to another person with an annual income of $40,000 [$80,000]. This person, whom we call the recipient, cannot make any decisions. The income of the recipient is clearly communicated to the allocator and vice versa. Both people are completely anonymous to each other and to the researchers.

The researchers give the allocator $100, and the recipient matched to him/her has not been given any money. The allocator has to decide now, how he would like to allocate the $100 between him/herself and the recipient. In particular, the allocator can send any amount between $0 and $100 to the recipient. The recipient is told that the decision-maker received $100, the choice the allocator had, and the decision made.

#### Comprehension questions

We would like to make sure that the key information has been clearly delivered to you. Please answer the following questions below before we proceed to the next stage.

1. What is the allocator’s annual income in USD? $_____ (correct answer depends on treatment)
2. What is the recipient’s annual income in USD? $_____ (correct answer depends on treatment)
3. How much money the allocator can distribute in USD? $ ____ (100).

#### Main questions

We are interested in your opinion now.

What amount, between $0 and $100, should the allocator send to the recipient:

$_____ (enter a number between 0 and 100).

Motivate why you think this is an appropriate amount:

[Participants are provided with the description of the scenario throughout the decision process]

#### Scoring of respondent motivations

Respondents provided open-ended motivations for the allocations they indicated as appropriate. For each respondent, we construct a variable *Motivation*, coded as 1 if the motivation provided by the respondent only contains the keywords related to inequality (and thus nothing related to fairness concerns). We use the following keywords to identify concerns over inequality: inequality, unequal, rich, poor, recipient needs, help, more, less, poverty, generous. If a respondent expressed both fairness and inequality concerns, we code the variable as 0. If the motivation text contains only “fair” as the keyword, we code it as − 1. Keywords are considered relevant as long as the string of the motivation text contains the keywords. For instance, if a respondent wrote the word “richer” instead of “rich”, we still count her as expressing inequality concerns. Respondents who expressed neither fairness nor inequality concerns are excluded from the motivation analysis.

### Study 3

#### Sample and design

The study was conducted with 837 U.S. participants through MTurk, following the same recruitment procedure as in Study 1. Respondents were randomly assigned to one of four conditions that elicit expectations regarding the previously collected behavior of a sample of millionaires’ in a simple allocation game conducted by Smeets and coauthors^7^: 197 participated in the empirical expectation with millionaire recipient condition; 205 participated in the empirical expectation with low-income recipient condition; 216 participated in the normative expectation with millionaire recipient condition; and 219 participated in the normative expectation with low-income recipient condition.

#### Instructions

The instructions of the survey experiment read as follows.

A recent study investigated the social behavior of wealthy individuals in Europe, who have more than 1 million euros in their bank account. These wealthy individuals were given 100 euros by the researcher, and were asked how much of the 100 euros they wanted to share with another person. Whatever they decided to give away was truly given to another person, and they kept the remaining amount. That is, the sharing decisions were implemented for real.

Importantly, the wealthy individuals making the sharing decision knew that the recipient paired with them was also wealthy, having more than 1 million euros in his/her bank account [was a low-income person who earned less than 12,500 euros per year before taxes].

All interactions were strictly anonymous and money payments and transfers were arranged by the researchers.

Many studies have looked at how people share an amount of money given to them in an experiment with an anonymous other person. Most of these studies involved neither particularly wealthy nor particularly low-income participants. The finding from these studies was that people on average give away about 28 percent of the given money to the other person.

##### Condition: empirical expectations, millionaire recipients

Do you think that the wealthy individuals in the study on average gave more or less than the typical share of 28 percent to the other person, when sharing with another wealthy person?

##### Condition: empirical expectations, low-income recipients

Do you think that the wealthy individuals on average gave more or less than the typical share of 28 percent to the other person, when sharing with a low-income person?

##### Condition: normative expectations, millionaire recipients

We are interested in what you consider an appropriate behavior by the wealthy individuals in this situation.

Should the wealthy individuals give more or less than the typical share of 28 percent to the other person, when sharing with another wealthy person?

##### Condition: normative expectations, low-income recipients

We are interested in what you consider an appropriate behavior by the wealthy individuals in this situation.

Should the wealthy individuals give more or less than the typical share of 28 percent to the other person, when sharing with a low-income person?

*Next, conditional on their first answer, the participants answered a question regarding the precise amount they have in mind:*

##### Condition: empirical expectations

You think the wealthy individuals gave more {less} to the other person than typically observed. How much of 100 euros do you think they gave on average to the other wealthy person [the low-income person]? *Respondent types in number between 28 and 100 {between 0 and 28}.*

##### Condition: normative expectations

You think the wealthy individuals should give more to the other person than typically observed. How much of the 100 euros do you think they should give to the other wealthy person [the low-income person]? *Respondent types in number between 28 and 100 {between 0 and 28}.*

### Supplementary Information

Supplementary Figure 1.

Supplementary Figure 2.

Supplementary Table 1.

## Data Availability

Data from Study 1 (US sample), Study 2, and Study 3 are available at Heidelberg University’s data repository at https://heidata.uni-heidelberg.de/dataverse/awiexeco. We have no rights to publish data from Study 1 (Chinese Sample). Shandong General Social Survey should be approached for any data issues.
