# Supplementary figures and images for: High-status individuals are held to higher ethical standards
Source: Sci Rep. 2023 Sep 13;13:15111. doi: 10.1038/s41598-023-42204-z (PMC10499905; doi:10.1038/s41598-023-42204-z)

Supplementary Figure 1. Average Appropriate Transfer by Provided Motivation

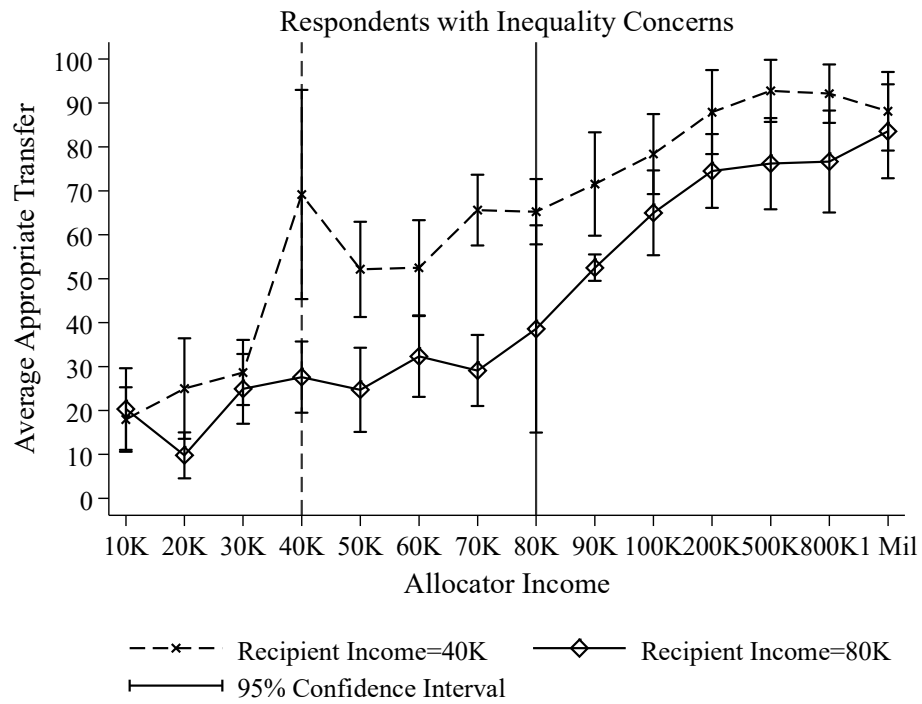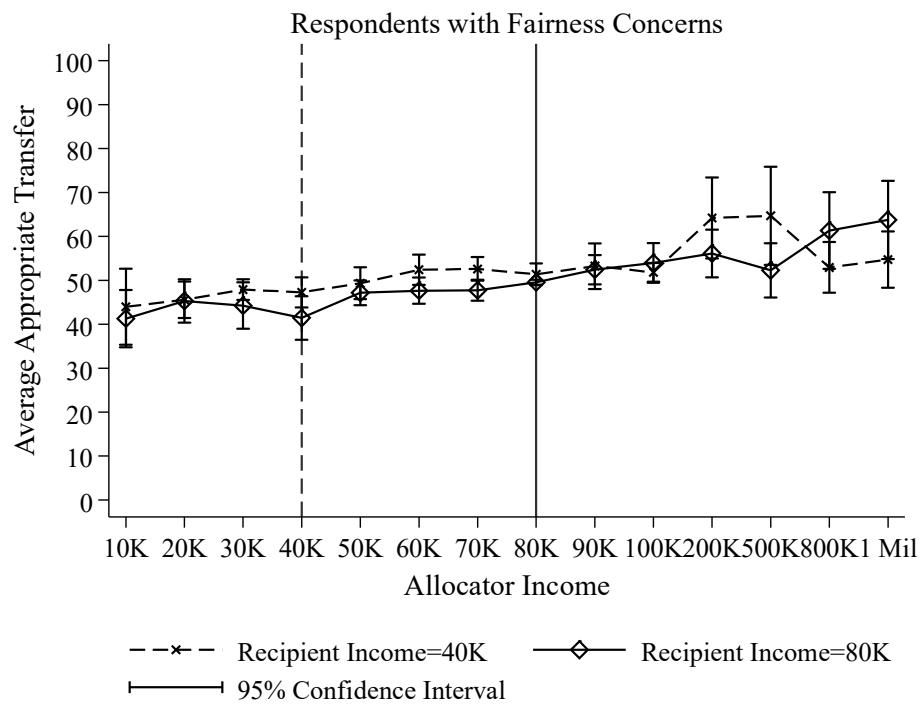

Supplement: Supplementary file 1 — Supplementary Figure 1. [file 41598_2023_42204_MOESM1_ESM.pdf]
