# Supplementary material for: High-status individuals are held to higher ethical standards
Source: Sci Rep. 2023 Sep 13;13:15111. doi: 10.1038/s41598-023-42204-z (PMC10499905; doi:10.1038/s41598-023-42204-z)
Supplement: Supplementary file 2 — Supplementary Figure 2. [file 41598_2023_42204_MOESM2_ESM.pdf]

Supplementary Figure 2. Allocator Income and Reported Motivations

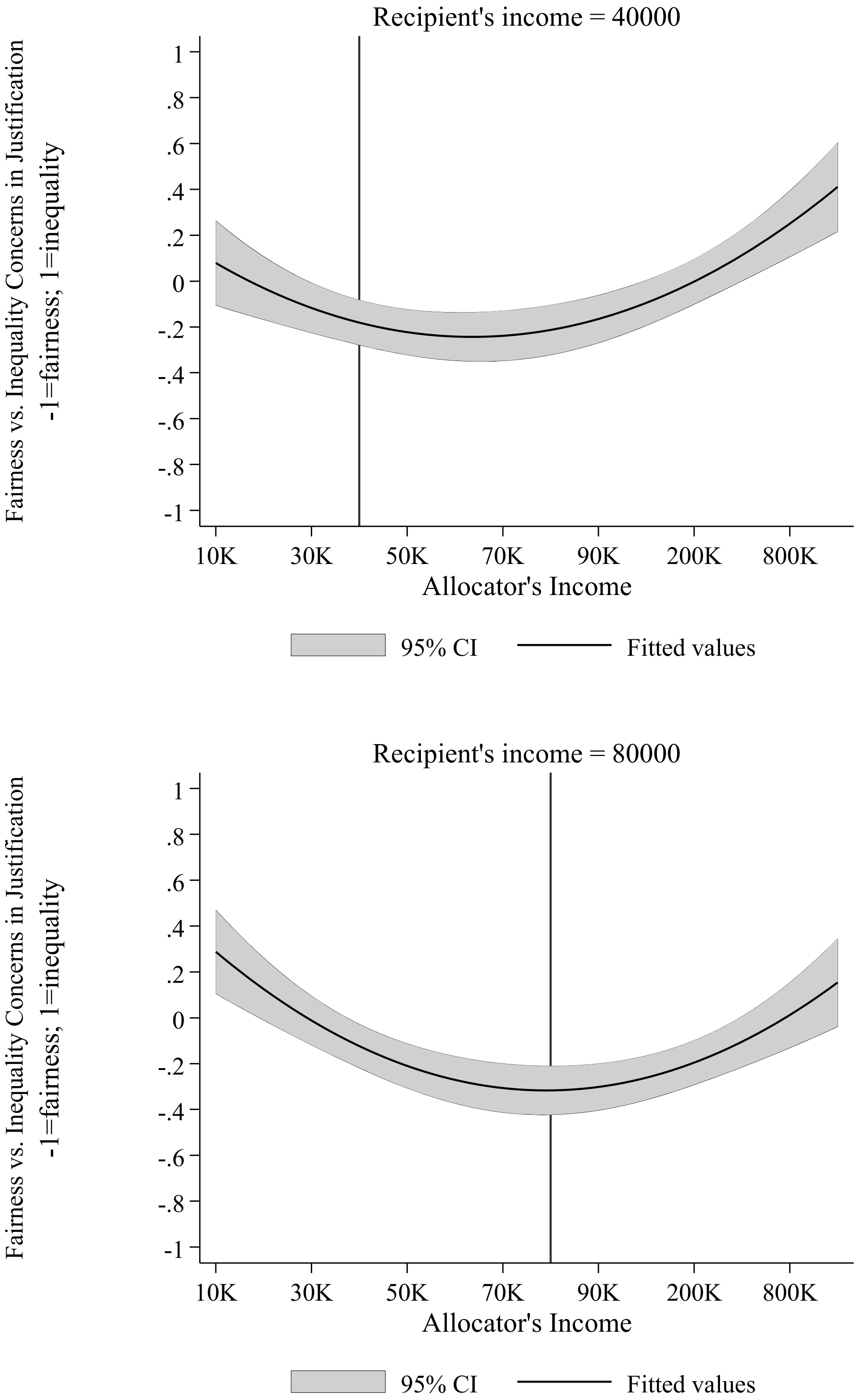

Notes: The figure shows the fitted values of a regression of the variable Motivation on allocator income level and income level squared (categorial variables). Both the linear and the quadratic terms are highly significant with p-value < 0.001 (robust standard errors) for both recipient income levels.
