# Supplementary material for: High-status individuals are held to higher ethical standards
Source: Sci Rep. 2023 Sep 13;13:15111. doi: 10.1038/s41598-023-42204-z (PMC10499905; doi:10.1038/s41598-023-42204-z)
Supplement: Supplementary file 3 — Supplementary Table 1. [file 41598_2023_42204_MOESM3_ESM.pdf]

Supplementary Table 1. Determinants of Appropriate Transfer

	Recipient Income $40,000 (1)	Recipient Income $80,000 (2)	Recipient Income $40,000 (3)	Recipient Income $80,000 (4)
Allocator Income	0.05***	0.05***	0.08***	0.07***
	(0.01)	(0.005)	(0.01)	(0.01)
High Family Income Respondent	1.31	9.28***	3.00	11.40***
	(2.90)	(2.79)	(4.03)	(3.69)
High Family Income Respondent × Allocator Income	-0.02**	-0.02***	-0.03**	-0.04***
	(0.01)	(0.01)	(0.02)	(0.01)
Male	-1.16	1.81	-2.26	2.50
	(2.16)	(2.12)	(3.20)	(2.94)
Age	-0.06	-0.10	-0.09	-0.2
	(0.09)	(0.08)	(0.13)	(0.11)
Constant	53.63***	42.46***	54.87***	40.27***
	(4.72)	(4.23)	(6.91)	(5.80)
# of Observations	548	591	548	591
(Pseudo) R^2	0.16	0.16	0.03	0.02

Notes: Dependent variable: transfer considered appropriate. Specifications (1) and (2) are OLS regressions and specifications (3) and (4) are Tobit regressions bounded from above ($100) and below ($0). Robust standard errors in parentheses: *** p<0.01, **, p<0.05, * p<0.1. The dummy variable *High Family Income Respondent* takes value 1 if the self-reported family financial situation is above or far above the average income in the area where the respondent lives in, and 0 if it is below or far below the average. Allocator income in $1,000.
